# Differences in Male Mate Recognition between the Invasive *Anoplophora glabripennis* (Coleoptera: Cerambycidae) and Japanese Native *A. malasiaca*

**DOI:** 10.3390/insects14020171

**Published:** 2023-02-09

**Authors:** Hiroe Yasui, Nami Uechi, Nao Fujiwara-Tsujii

**Affiliations:** Institute for Plant Protection, National Agriculture and Food Research Organization (NARO), Tsukuba 305-8666, Ibaraki, Japan

**Keywords:** sympatric species, mating behavior, contact sex pheromones, invasive species, native species

## Abstract

**Simple Summary:**

The invasive Asian longicorn beetle *Anoplophora glabripennis* and native Japanese white-spotted longicorn beetle *A. malasiaca* show an extensive overlap with host plants, niches, and emergence season. Hybridization between these two species is suspected. The surface of the female body is covered with contact sex pheromones that elicit male mating behavior within species. We evaluated the contact pheromonal activity of crude extract and fractions of female *A. glabripennis* and revealed a hydrocarbon fraction to show activity; however, it was relatively weak. There might be other, as yet unidentified active compounds in the extract. Only a small number of male *A. glabripennis* showed mating behavior when they were exposed to the crude extract of female *A. malasiaca*, whereas a considerable number of male *A. malasiaca* showed mating behavior when exposed to both female *A. glabripennis* and *A. malasiaca* extracts. Gomadalactones are essential contact pheromone components that elicit mating behavior in male *A. malasiaca*. We, therefore, attempted to detect gomadalactones in female *A. glabripennis* extract, but without success. We investigated the possible reasons for this phenomenon and the difference in male mate recognition systems between *A. glabripennis* and *A. malasiaca.*

**Abstract:**

The Asian longicorn beetle *Anoplophora glabripennis* is a recently arrived invasive species to Japan. The Japanese native *A. malasiaca* shows an extensive overlap with *A. glabripennis* with host plants, niches, and emergence season. Hybridization between these two species is suspected in Japan. The surface of the female is covered with contact sex pheromones that elicit male mating behavior within species. We evaluated the contact pheromonal activity of crude extract and fractions of female *A. glabripennis* coated on a black glass model and revealed a hydrocarbon fraction and a blend of fractions to show activity but relatively weak, suggesting the presence of other unknown active compounds. Few male *A. glabripennis* showed mating behavior when they were exposed to a crude extract of female *A. malasiaca*. However, a considerable number of *A. malasiaca* males mounted and showed abdominal bending behavior when presented with glass models that were coated with each extract of female *A. glabripennis* and *A. malasiaca*. Gomadalactones are essential contact pheromone components that elicit mating behavior in male *A. malasiaca*; however, we could not detect them in female *A. glabripennis* extract. Here, we investigated the possible reasons for this phenomenon and the difference in male mate recognition systems between these two species.

## 1. Introduction

The Asian longicorn beetle *Anoplophora glabripennis* (Motschulsky) (Coleoptera: Cerambycidae) is native to China and throughout Korea [1]. It has invaded and become established in several US states and several European countries, including Italy, Switzerland, France, and Germany, where attempts are being made to eradicate them [2,3]. It has also been eradicated in the United Kingdom, Finland, Austria, and Canada [2]. There were no reports of its establishment in Japan until 2021, in spite of it having invaded and been eradicated several times before 2003 [2]. It is, however, thought to have recently invaded and rapidly established itself in eight prefectures as of 2022 [4,5,6,7,8,9,10,11]. *Anoplophora grabripennis* has already caused substantial damage to street trees, mainly *Cercidiphyllum japonicum* (katsura)*, Ulmus parvifolia* (Chinese elm)*, Aesculus turbinata* (Japanese horse chestnut), and *Salix* spp. (willow).

This species is reported to infest trees in the genus *Prunus*, *Pyrus*, and *Malus* (Rosaseae), which includes several economically important fruit trees such as plums, pears, and apples [1,2,3,12]. The developing larvae of this beetle feed by boring inside the trunks and branches, causing damage that frequently kill the host trees. Its potential danger to economically important fruit production is highlighted by its recent invasion of Japan, following the invasive red-necked longicorn beetle (*Aromia bungii*), which is causing serious damage to stone fruit trees in Japan [13,14,15,16,17,18]. As the developing larvae are hidden inside their hosts, this beetle is difficult to control with insecticides.

Japan is a region in which the native white-spotted longicorn beetle, *Anoplophora malasiaca* is already causing substantial damage [2]. *Anoplophora malasiaca* is synonymized with *A. chinensis* by Lingafelter and Hoebeke in 2002 [1]; however, Makihara [19,20] stated that they are independent species. In this study, we use “*A. malasiaca”* as Japanese native species. *Anoplophora malasiaca* and invasive *A. glabripennis* are similar in appearance. This close resemblance prevented people in Japan from identifying *A. glabripennis* as an alien species.

Their host trees and niches partially overlap [12,21,22]. One of the authors (HY) caught adults of *A. glabripennis* and *A. malasiaca* at the same time on the same *C*. *japonicum* tree. Both adults are also found on willow trees (*Salix* spp). In the field, infestations by which species can be identified by the locations of their exit holes in trunks or branches: holes in trunks near the ground are made by *A. malasiaca*, and those in the upper parts of tree trunks in branches are made by *A. glabripennis*. Even if they utilize the same tree, their larval niches inside of the tree are likely to be separately distributed. The adult emergence period is almost the same, so they are highly likely to encounter each other on host trees. If they mate, reproductive disturbance might occur. *Anoplophora glabripennis*, *A. malasiaca,* and the related *A. chinensis* have been reported to have common volatile pheromones [23,24,25,26,27], whereas no common contact pheromone compounds have been reported [26,28,29,30,31,32,33,34]. Wang and Keena [35] investigated the hybridization potential among *A. glabripennis, A. chinensis,* and *A. malasiaca* using 4-week laboratory crossing experiments. Egg laying, hatching, and development were observed. The experiments revealed less than 100% conspecific pairings. Mating behavior between *A. glabripennis* and *A. malasiaca* was observed in 4 of 25 *A. glabripennis* female–*A. malasiaca* male pairs (16%) but in none of the 25 *A. malasiaca* female–*A. glabripennis* male pairs (0%). Their experiments revealed that *A. malasiaca* males will mate with *A. glabripennis* females and they lay eggs that hatch (at a rate of less than 10%) that develop into adults. If it happens in the field, it will become reproductive disturbance and have an impact on ecosystems. Sunamura et al. [36] reported mating behavior between *A. glabripennis* and Japanese *A. chinensis* (=*A. malasiaca*) caught in Japan. *Anoplophora malasiaca* males mounted *A. glabripennis* females and attempted to insert their genitalia. Both reports indicate the possibility of producing hybrid offspring. These observations also indicate that one or more mating recognition cues for *A. malasiaca* males are present on the female surface of *A. glabripennis*.

To clarify the chemical ecology of cerambycid beetles, we have reported the identification of the female contact pheromone of *A. malasiaca* [28,29,30,32,37,38]. The elytra of female *A. malasiaca* carry the pheromone compounds belonging to three chemical groups. Two are female-specific aliphatic hydrocarbons and ketones, and the third group, which is essential to elicit male mating behavior, consists of three lactones: gomadalactones A, B, and C. When individual gomadalactones or a mixture of them all were blended with hydrocarbons and ketones at the same doses as those that were found in female extract, males showed mating behavior [32]. The identification of female contact pheromone compounds in *A. glabripennis* was carried out by Zhang et al. [33]. They identified five alkenes: (*Z*)-9-tricosene, (*Z*)-9-pentacosene, (*Z*)-7-pentacosene, (*Z*)-9-heptacosene, and (*Z*)-7-heptacosene. Individually, the compounds were inactive, but the blend of all five elicited mating behaviors from males, however, they were lower than those from crude extracts from females. These pheromonal hydrocarbons are completely different from those of *A. malasiaca* contact pheromone components; however, some individuals can mate with different species. We report herein on chemicals that may elicit mating behavior in other species and discuss the potential for hybridization of these related species. It is highly likely that there are as yet unknown “pseudo” contact sex pheromone compounds on the elytra of female *A. glabripennis* that elicit mating attempt in males of *A. malasiaca.*

## 2. Materials and Methods

### 2.1. Collection of Insects

The two Anoplophora species can be distinguished by appearance. *Anoplophora malasiaca* adults of both sexes have the characteristic of possessing 20–40 small projections (tubercles) on the basal one fifth of each elytron. This characteristic can allow *A. malasiaca* to be distinguished from *A. glabripennis* [1,2]. *Anoplophora glabripennis* adults were collected by hand from *Katsura* (*Cercidiphyllum japonicum*) and willow (*Salix* spp.) trees in Tsukuba, Ishioka, Omitama, and Sendai Cities from mid-June to July 2021 and 2022. A total of 40 individuals were collected. *Anoplophora malasiaca* adults were collected by hand from a rose (Rosaceae) garden in Ishioka, willow trees in Jôsô and Tsukuba Cities in late June to July, and from mandarin orange (*Citrus unshiu* Marc.) groves on Kunisaki Peninsula, Oita Prefecture, Japan, in mid-July 2022. Beetles were individually reared in clear plastic cups (~11 cm diam. × 9.5 cm height) at 24 °C under a 15L:9D photoperiod, illuminated by fluorescent lamps. Each *A. glabripennis* beetle was fed willow and *C. japonicum,* and *A. malasiaca* was fed willow and citrus branches, replaced every five days. All of the cut branches were stored at 5 °C and used within 10 days.

### 2.2. Extraction of Female Contact Pheromone

Females of both species were frozen and stored at −30 °C. The elytra of females were removed and placed in diethyl ether (1.5 mL/female). After 5 min at room temperature, the extract was decanted. The elytra were rinsed twice with ether, and the rinses were added to the extract. The ether was removed from the extracts under reduced pressure at <30 °C, and the resulting residue (“female extract”) was dissolved in *n-*hexane (hexane) and stored at −30 °C until use. Keeping extracts in diethyl ether, unexpected peroxide production may occur. We confirmed no residue of the extracts was found as hexane solution. The solvents that were used were HPLC grade.

### 2.3. Fractionation of Female Extract

Female elytra extract of *A. glabripennis* (4 female equivalent, fe) was fractionated using silica gel column chromatography (Wakogel C-200, 0.5 g, Fujifilm-Wako, Richmond, VA, USA). Each 5-mL aliquot of hexane, ether, and ethyl acetate (AcOEt) eluent was concentrated and stored at −30 °C until use. Female elytra extract of *A. malasiaca* was also fractionated in a same manner for providing chemical analysis.

### 2.4. Abdominal Bending Assay (Bioassay 1) (Figure 1a)

Behavioral activity was evaluated as in Fukaya et al. [39], between 13:00 and 17:00 (9–13 h after lights on). A capsule-shaped black glass model (12 mm diam. × 35 mm length) was used as a female model. It was attached to the center of a filter-paper disc and coated with the test material (0.5 fe) dissolved in ca. 20 μL hexane. A male beetle was placed nearby and allowed to touch the model with his antennae. The following responses were noted: the male placed his forelegs on the model (=holding), the male grasped the model with his fore- and mid-legs and adjusted his body axis to that of the model (=mounting), and the male bent his abdomen downward along the model surface (=abdominal bending). Sexual maturity of the males was confirmed by observation of abdominal bending behavior toward intact females before tests. Each individual was used once per day. The sample sizes of males were 31 for *A. malasiaca* and 44 for *A. glabripennis*.

### 2.5. Recording Male Behavior to Evaluate Holding Activity When Exposed to Female Extract and Fractions (Bioassay 2) (Figure 1b)

Behavioral assays were conducted between 09:00 and 17:00 at 25 °C (4 h recording, 4 h after lights on). The black glass model shown above was used as a female model. It was affixed with a small piece of adhesive tape to the center of a filter paper disc (15 cm diam., Toyo No. 2, Toyo Roshi Kaisha, Tokyo, Japan) and coated with female extract or fraction (0.2 fe), dissolved in ca. 20 μL hexane or AcOEt. A male beetle was placed near a model coated with female extract or solvent-only and covered with a clear plastic cup (the same as used to rear them). We tested 4 or 5 males separately at the same time. Male behavior was recorded every 5 min using a time-lapse camera. When a male was seen to be holding the model, it counted as positive. The frequencies of holding were compared. Due to the limitation of samples, bioassays were performed with 0.2 fe, which was the least amount but could induce male positive response. The sample sizes were 10 to 14.

### 2.6. GC/MS Analyses

For the hydrocarbon fractions, GC/MS analyses were performed on an Agilent 7890A GC system (Santa Clara, CA, USA) interfaced to a JMS-T100GC Time-of-Flight Mass Spectrometer (JEOL, Tokyo, Japan) in EI mode with 70 eV at 220 °C. Injection was set for splitless mode at 220 °C for 1 min. A DB-5MS capillary column (30 m × 0.25 mm ID × 0.25 μm film thickness; Agilent) was used. Helium was the carrier gas, and constant flow mode 1.1 mL/min. The GC oven temperature program was held for 1 min at 40 °C, increased from 40 to 280 °C at 10 °C min^−1^, and held at 280 °C for 10 min. Compounds were identified by comparison with the mass spectra of major peaks as reported by Fukaya et al. [28].

Unsaturated components were derivatized to decide the position of double bonds. After separation by silver nitrate impregnated silica gel column chromatography, epoxidation was performed by *m*-chloroperbenzoic acid.

### 2.7. High Performance Liquid Chromatography (HPLC)

The EtOAc fraction was analyzed on a 10ADVp system HPLC (Shimadzu, Nakagyo-ku, Kyoto, Japan) equipped with a Cosmosil Cholester column (4.6 mm ID × 250 mm, Nacalai Tesque, Kyoto, Japan) at room temperature. The mobile phase was 45% methanol in water. The flow rate was 1 mL min^−1^. Peaks were monitored by UV-absorbance at 210 and 254 nm and were identified by comparison with those of authentic compounds [32]. The synthetic three gomadalactones A, B, and C were eluted at 11.6, 14.7, and 15.4 min. Two female-equivalents (fe) of the extract were needed to perform one HPLC analysis.

### 2.8. Statistical Analysis

Individuals showing an abdominal bending response were analyzed using Pearson’s Chi-squared test with Yates’ continuity correction. The frequencies of males holding onto the female model were analyzed by GLM. The probability distribution of the response variable was a Poisson distribution, and a log function was specified as the link function. Frequencies were analyzed by one-way analysis of variance (ANOVA), followed by Tukey’s multiple comparison test with Bonferroni’s correction at *p* < 0.05. Statistical analyses were performed using R version 4.1.0 [40].

## 3. Results

### 3.1. Male Copulation Responses to Glass Models Coated with Female Extracts of Two Different Anoplophora Species (Bioassay 1)

**Figure 1:**
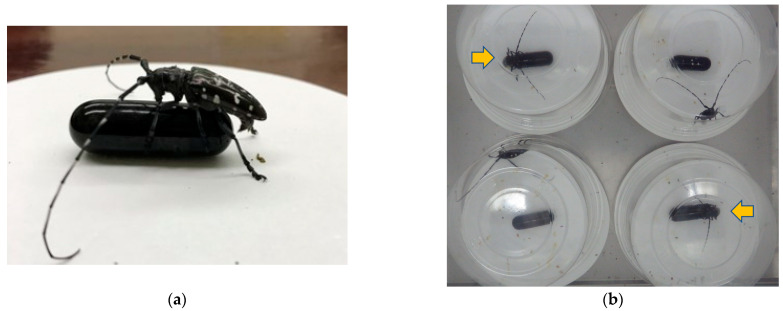
*Anoplophora glabripennis* males. (**a**) A male persistently held and bent his abdomen toward a glass model that was coated with conspecific female extract (Bioassay 1); and (**b**) males with glass models that were coated with the female extract. Two of them (yellow arrows) persistently held and bent their abdomens toward the models (Bioassay 2).

**Figure 2:**
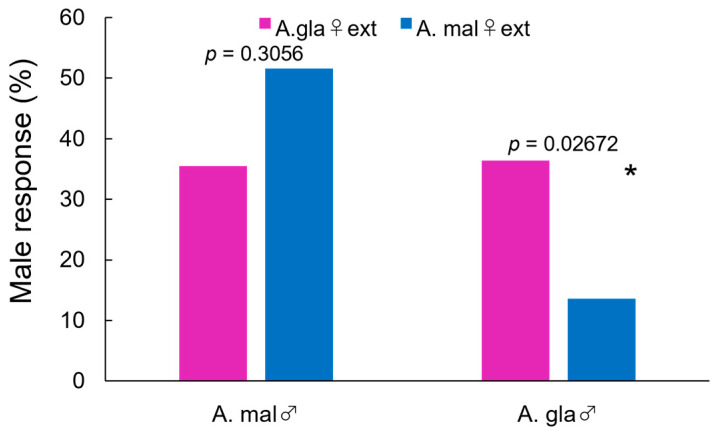
Male abdominal bending response to a glass model that was coated with *A. glabripennis* or *A. malasiaca* female extracts (0.5 fe) (Bioassay 1). N = 31 for *A. malasiaca* males, and N = 44 for *A. glabripennis* males. * Data were analyzed using Pearson’s Chi-squared test with Yates’ continuity correction at *p* < 0.05.

When a glass model that was coated with female extract was presented to the males, they sometimes showed holding behavior followed by abdominal bending (Figure 1a). Copulating activity level of male *A. malasiaca* toward the female extract of *A. glabripennis* that was comparable (36%) to that which was induced by conspecific female extract (52%) was confirmed (*χ*^2^ = 1.0497, *df* = 1, *p* = 0.3056, Figure 2). In contrast, female extracts of *A. malasiaca* induced behavioral responses in only 14% of male *A. glabripennis*: significantly less than conspecific female extract (36%; *χ*^2^ = 4.9091, *df* = 1, *p* = 0.0267). Due to the low rates of responding males, this bioassay method was not used in subsequent bioassays to compare the pheromonal activities of fractions from female extract.

### 3.2. Frequency of Mate-Holding of Glass Models Coated with Female Extracts of Two Different Anoplophora Species (Bioassay 2-1)

During 4 h of recording, some males held onto the model for a long time. Although some held onto it for a shorter time and left it, they returned to it and held it again (Figure 1b). Males that were presented with a solvent-only model walked around on the floor and on the wall of the plastic cup.

**Figure 3:**
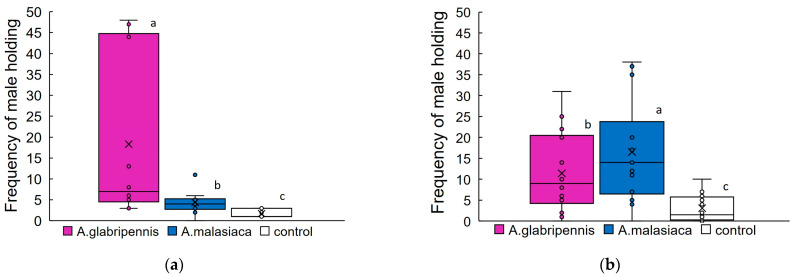
Box-plots of frequencies of (**a**) *A. glabripennis* males holding onto glass models that were coated with *A. glabripennis* or *A. malasiaca* female extracts (Bioassay 2, N = 10); and (**b**) *A. malasiaca* male holding onto glass models that were coated with *A. glabripennis* or *A. malasiaca* female extracts (Bioassay 2, N = 14). Female extracts were coated with 0.2 female equivalent. Control: glass models that were coated with solvent only. Box-plots: middle line, mean; boxes, 75 and 25% quartiles; x, average; whiskers, maximum and minimum values; o, outliers. *p* < 2.2 × 10^16^, GLM, Poisson (log). Frequencies were analyzed using Tukey’s multiple comparison test with Bonferroni’s correction at *p* < 0.01. Different letters indicate significant differences.

Although there was large discrepancy in variation between individual data points, male *A. glabripennis* held onto glass models that were coated with conspecific female extract significantly more frequently (18.3 ± 6.2; mean ± SE, per 48 observations, N = 10; Figure 3a) than with female extract of *A. malasiaca* (4.4 ± 0.9, N = 10). The frequency of holding of solvent-only models was 1.7 ± 0.4 (N = 10), lower than for models that were coated with female extracts.

Male *A. malasiaca* held onto glass models that were coated with conspecific female extract significantly more frequently (16.5 ± 3.3, N = 14; Figure 3b) than with female extract of *A. glabripennis* (11.4 ± 2.6, N = 14). The frequency of holding to solvent-only models was 3.1 ± 0.9, significantly lower than for models that were coated with female extracts.

### 3.3. Frequency of Male A. glabripennis Holding onto Glass Models Coated with Fractions of Conspecific Female Extracts (Bioassay 2-2)

**Figure 4:**
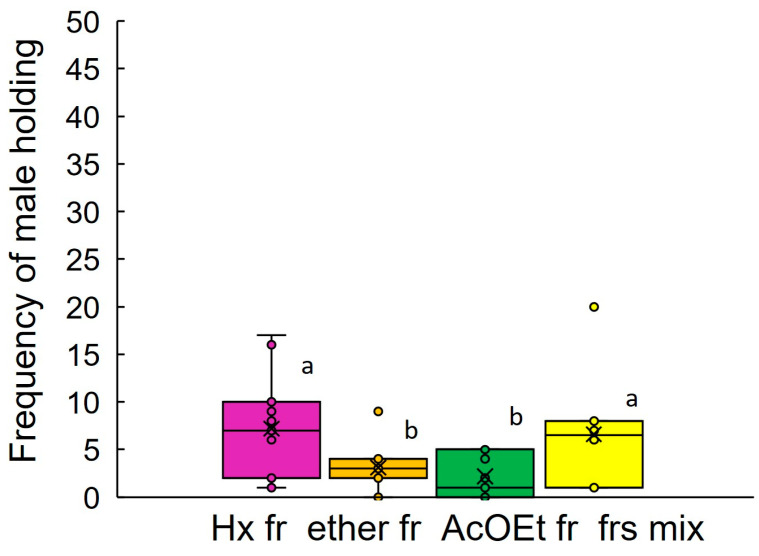
Box-plots of frequencies of *A. glabripennis* males holding onto glass models that were coated with fractions of conspecific female extracts (0.2 fe) (Bioassay 2). Hx: Hexane fr., ether: diethyl ether fr., AcOEt: ethyl acetate fr., frs. mix: blend of three fractions. 0.2 fe. N = 10–11. Box-plots: middle line, mean; boxes, 75 and 25% quartiles; x, average; whiskers, maximum and minimum values; o, outliers. *p* < 2.2 × 10^16^, GLM, Poisson (log). Frequencies were analyzed using Tukey’s multiple comparison test with Bonferroni’s correction at *p* < 0.05. Different letters indicate significant differences.

Female extract of *A. glabripennis* was fractionated into three fractions using silica gel column chromatography. Males of *A. glabripennis* held onto glass models that were coated with the hexane fraction of the extract (7.2 ± 1.7, N = 11) (Figure 4) more frequently than the other two fractions: the ether fraction (3.1 ± 0.7, N = 11) and AcOEt fraction (2.2 ± 0.6, N = 11), and comparable to a blend of the three fractions (6.6 ± 1.8, N = 10). The frequency of males holding to the hexane fraction was, however, relatively low.

### 3.4. Hydrocarbons in Hexane Fractions of Female Extracts

**Figure 5:**
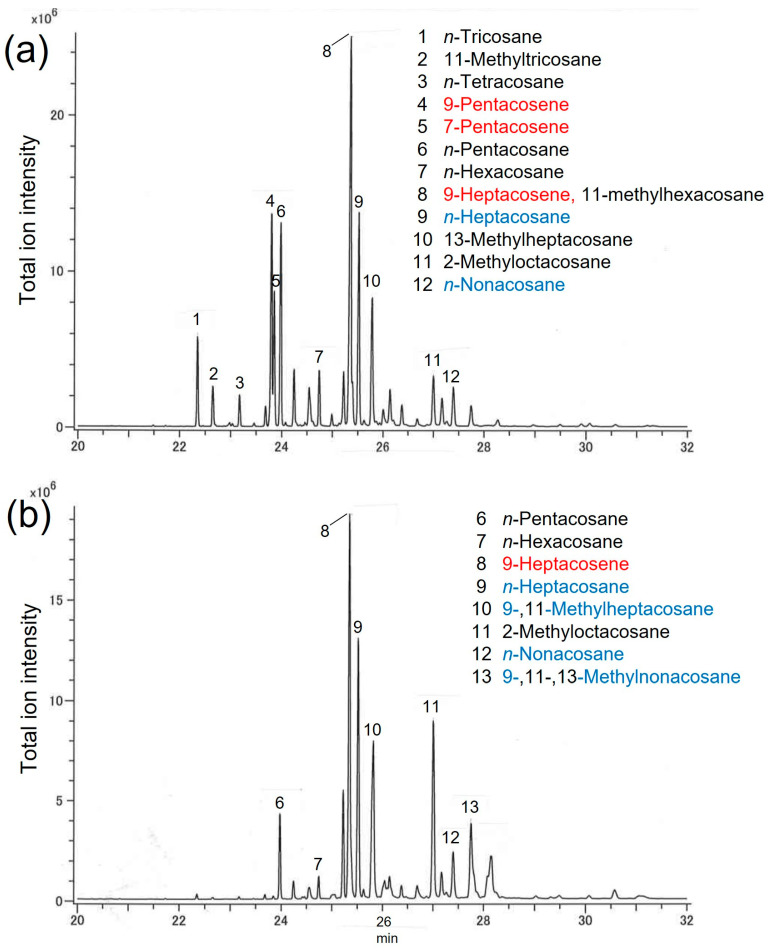
GC profiles of hexane fractions of female extract of (**a**) *Anoplophora glabripennis*, and (**b**) *A. malasiaca.* Olefin geometries were not determined. Compounds written in red are contact pheromone components of *A. glabripennis* and these in blue are those of *A. malasiaca*.

Hydrocarbons were analyzed by GC/MS (Figure 5). The positions of methyl branch of hydrocarbons were detected by diagnostic fragmentation ions of their mass spectra. The positions of double bonds were detected by epoxidated hydrocarbons with GC-MS analysis. Females of *A. malasiaca* had a higher proportion of longer chain hydrocarbons above C27 than did *A. glabripennis* females (*A. malasiaca*: over 80% and *A. glabripennis*: 50% of total C23–C33). The compositions of the hydrocarbon fractions of *A. malasiaca* females and *A. glabripennis* females have some components in common (*n*-pentacosane, *n*-hexacosane, 9-heptacosene, *n*-heptacosane, 2-methyloctacosane, *n*-nonacosane: Figure 5). We detected three alkenes that were reported as contact pheromone components [33] in the hexane fraction of female elytra extract of *A. glabripennis* that were collected in Japan. Whereas four contact pheromone compounds were detected in *A. malasiaca* extract [28]. In *A. glabripennis* extract, two pheromone components of *A. malasiaca* were detected, and conversely, one pheromone component of *A. glabripennis* was detected in *A. malasiaca* extract.

### 3.5. HPLC Analysis of AcOEt Fractions of Female Extracts

The EtOAc fractions of *A. glabripennis* and *A. malasiaca* female extracts were analyzed by HPLC and compared with synthetic gomadalactones. The fraction of *A. malasiaca*, three peaks were detected with a UV detector and the retention times and UV spectra matched to synthetic gomadalactones A, B, and C, respectively. Whereas, in the EtOAc fraction of *A. glabripennis*, an unknown broad peak was detected at 16.1 min with UV 210 nm, and a small peak at 11.6 min with both 210 and 254 nm. We tried to isolate compounds in *A. glabripennis* female by HPLC with three different columns, however, because of the limited amount of extract, we could not perform this yet. We could not detect any peaks by GCMS analysis in neither the AcOEt fractions nor synthetic gomadalactones.

## 4. Discussion

We first compared the abdominal bending behavior of males in response to glass models that were coated with female extracts of *A. malasiaca* and *A. glabripennis* (Bioassay 1). Males of *A. glabripennis* bent their abdomen significantly more to models that were coated with conspecific female extract (36%) than to those with *A. malasiaca* female extract (14%), whereas males of *A. malasiaca* showed no significant difference in response between the two species (52% to *A. malasiaca* and 35% to *A. glabripennis*). This result supports the results of Sunamura et al. [36] who reported that approximately 50% of Japanese *A. chinensis* (=*A. malasiaca*) males attempted to copulate with *A. glabripennis* females. They also support an account that confirmed mating behavior in four *A. glabripennis* female–*A. malasiaca* male pairs (16%, N = 25) and none of the *A. malasiaca* female–*A. glabripennis* male pairs (0%, N = 25) by Wang and Keena [35].

The levels of mating attempts to crude extracts of conspecifics were 52% and 36%, both seems rather low compare to our previous reports [31]. In this study, we collected beetles from several areas and host plants, so the differences of their backgrounds may affect the relatively low response rate. Adult *A. glabripennis* were also fed willow and *C. japonicum*, and *A. malasiaca* adults were fed willow and citrus branches, so we cannot rule out the possibility that these different food sources affected their responses [31,41,42]. We had observed high levels of mating attempts within same host plant populations, conversely, lower response rate between different host plant populations in *A. malasiaca* [31]. We do not believe there to have been a significant effect, since we observed no particular rejection responses in this study, even in extracts from females that were fed citrus branches; the male response was either no response or holding [42].

When we applied the abdominal bending assay (Bioassay 1) to evaluate the activity of the fractions of female extract of *A. glabripennis*, the response rates were insufficient to permit statistical analysis. A similar response tendency or a more sensitive evaluation was obtained in this study for frequencies of males holding the glass models that were coated with female extracts of both species (Bioassay 2). This assay method can also be used to evaluate the activity of fractions from extracts. We, therefore, carried out fractionation of female extract of *A. glabripennis*. Since the hexane fraction of the female extract was more active than the other fractions, and the activity did not increase when the other fractions were mixed with the hexane fraction, the contact pheromone of this species might be present only in the hexane fraction, which would agree with the report by Zhang et al. [33]. The hexane fraction of female extract of *A. glabripennis* in this study contained three of five pheromone compounds that were identified by Zhang et al. [33] using GC-MS analysis (Figure 5). However, their identification of contact pheromones was based on the sex-differentiated hydrocarbons, so it would not be surprising if there were other compounds with contact pheromone activity. The activity of the hexane fraction was relatively low, suggesting there to be other active components. These could be unstable compounds, or the active compounds could appear in more polar fractions; they would likely have appeared after the AcOEt eluent. Performing EAG experiments is considered to confirm candidate compounds generally, however, contact pheromone components have low volatility and it is difficult to apply these in EAG experiments.

The reason for most *A. glabripennis* males exhibiting no mating behavior in the presence of *A. malasiaca* females is that *A. glabripennis* males did not interpret the components on *A. malasiaca* females as being conspecific contact pheromones. The compositions of the hydrocarbon fractions of *A. malasiaca* females and *A. glabripennis* females have some components in common (Figure 5). Male *A. glabripennis* are reported to be unresponsive to each of the five pheromone hydrocarbon components individually, which are abundant on the female body surface, but react to a blend [33]. This report suggests that one pheromone component, 9-heptacosene, which was found on the surface of female *A. malasiaca*, were insufficient for male *A. glabripennis* to respond.

**Figure 6:**
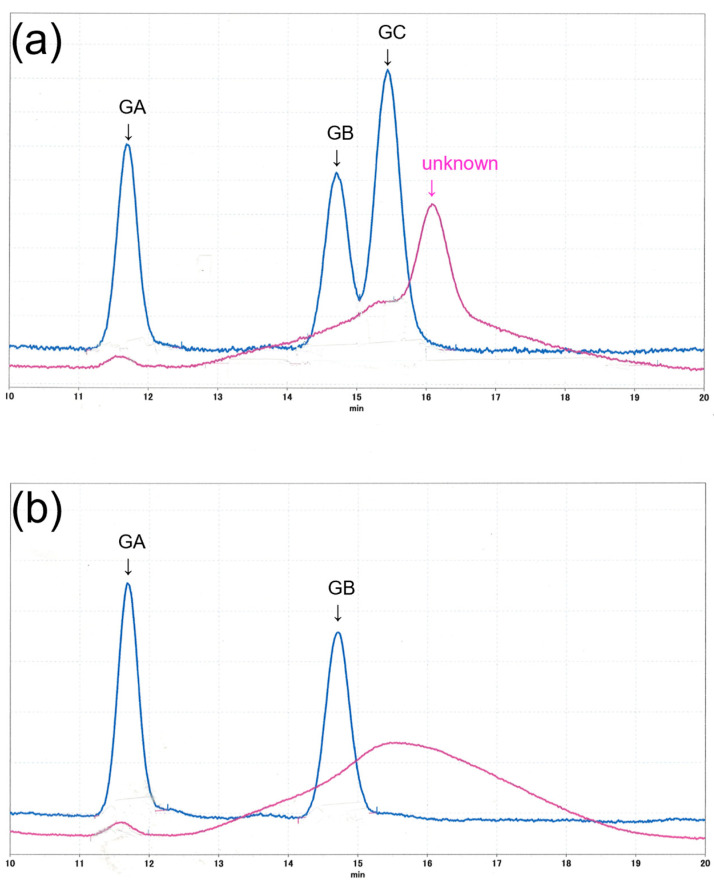
HPLC profiles of ethyl acetate fractions of female extracts of *A. glabripennis* (red lines, 2 fe) and *A. malasiaca* (blue lines, 0.2 fe). (**a**) detected by UV 210 nm, and (**b**) 254 nm. GA: gomadalactone A; GB: gomadalactone B; GC: gomadalactone C.

Why did a surprising proportion of *A. malasiaca* males show mating behavior upon encountering *A. glabripennis* females? We hypothesized that the body surface of *A. glabripennis* female contains active components that cause *A. malasiaca* males to identify her as a conspecific female. When applied alone, the AcOEt fraction evoked weak male mating behavior, but removal of the fraction from the mixture (=hydrocarbon components and ketone components) caused a complete cessation of pheromonal activity. Thus, the fraction containing gomadalactones is essential for evoking precopulatory behavior in male *A. malasiaca* [32]. We are convinced that some response by male *A malasiaca* to *A. glabripennis* extract was never caused by hydrocarbon components on *A. glabripennis* females. We, therefore, performed HPLC analysis of the AcOEt fraction of the female extract of *A. glabripennis* that was suspected to contain gomadalactones, essential components of contact pheromones of *A. malasiaca* [30] (Figure 6). The AcOEt fractions of female extracts of *A. glabripennis* and *A. malasiaca*, and synthetic gomadalactones A, B, and C, were also analyzed simultaneously for comparison. The fraction of *A. malasiaca* had three peaks with the same retention times as the synthetic gomadalactones, and the UV spectra were also consistent with the synthetic ones. Gomadalactones were originally isolated from *A. malasiaca* of same population by our group, confirming that those are gomadalactones. The retention time of *A. glabripennis* had a trace peak with the same retention time as that of gomadalactone A, but the retention times of B and C were masked by a broad peak, so B and C could not be detected. Interestingly, a relatively large peak was observed immediately after the retention time of C, with UV absorption that was similar to that of gomadalactone C, suggesting it to be an analog of C. Unfortunately, we were unable to obtain a sufficient amount of this unknown component to be able to measure the NMR spectra needed to determine its structure. The *A. malasiaca* female that was used in this study had approximately equal amounts of gomadalactones A, B, and C. The recognition of gomadalactones by *A. malasiaca* males is not very specific, and activity is observed for A, B, and C individually as well as for their analogues [32]. We had tested several gomadalactone derivatives such as gomadalactone C diastereomer and revealed most of them had contact pheromonal activity as same level as gomadalactone A, B, and C [32]. Therefore, we supposed gomadalactone derivatives must be included in the extract of *A. glabripennis* female. *Anoplophora malasiaca* males may recognize *A. glabripennis* females as sympatric females if the *A. glabripennis* female carries a gomadalactone analog. We intended to carry out contact pheromone assays with the extract and fractions of *A. glabripennis* females using *A. malasiaca* males, but we were not able to collect enough *A. glabripennis* females for bioassays, so this will be a future study.

Wang and Keena [35] conducted their experiments using the following individuals: an *A. glabripennis* colony was established from beetles that were collected in 1999 in Chicago. The *A. malasiaca* colony used consisted of 16th, 17th, and 18th laboratory generations of an invasive Italian population, captured in Japan. Therefore, we are not sure that their behavior is identical to that will be shown by wild beetles. Even though their experiments revealed that *A. malasiaca* males will mate with *A. glabripennis* females and they lay eggs that hatch at a rate of less than 10% that develop into adults, possibly facilitating asymmetric hybridization. These species-specific features of invasive beetles have an impact on their adopted ecosystems and cause economic concerns. An investigation is needed into whether hybrid offspring are capable of continued reproduction.

## Data Availability

Data are available upon request.
